# Preliminary functional results after transanal irrigation in patients undergoing SHiP procedure for low rectal cancer

**DOI:** 10.1007/s13304-022-01334-1

**Published:** 2022-07-18

**Authors:** Francesco Bianco, Sebastiano Grassia, Marta Goglia, Gaetano Gallo

**Affiliations:** 1 Department of Surgical Sciences, “Sapienza” University of Rome, Rome, Italy; 2 Department of Surgery, Sant’Andrea Hospital, Sapienza University of Rome, Via di Grottarossa 1037, 00189 Rome, Italy; 3 General Surgery Unit, S. Leonardo Hospital, Castellammare Di Stabia, Naples, Italy

**Keywords:** Transanal irrigation (TAI), Short-stump and high-anastomosis pull-through (SHiP) procedure, Low anterior resection syndrome, Rectal cancer, Functional disorders

## Abstract

The short-stump and high-anastomosis pull-through procedure (SHiP) is a newly introduced technique in the treatment of rectal cancer. This procedure does not involve the creation of a diverting ostomy with great improvement of the patients’ quality of life in the post-operative period. However, functional post-operative alterations such as low anterior rectal resection syndrome (LARS) may occur. In this context, trans-anal irrigation (TAI) may represent a viable option in the treatment and management of LARS symptoms. The aim of the present study is to investigate the role of TAI in patients operated on SHiP procedure for low rectal cancer. A prospective database of 17 patients who underwent a SHiP procedure was maintained from April 2019 to December 2021. Anal continence and functional outcomes were assessed through LARS score and Cleveland Clinic Incontinence Score (CCIS), respectively. All patients with a LARS score > 21 underwent TAI in the post-operative period. LARS median value was 36 (IQR = 8) and drastically improved after TAI treatment to 3 (IQR = 3), as the CCIS at a mean follow-up of 9 months (SD ± 5.02). Good functional result was reached in 12 out of 13 patients (92%). Our study confirms that patients with severe post-operative dysfunction could benefit from the use of TAI.

## Background

Rectal surgery has recently become a talking point regarding the choice of the most appropriate surgical technique in case of patients with low rectal cancer. Traditional colo-anal anastomosis with diverting ostomy has to be compared nowadays with a recently modified Turnbull–Cutait (TC) technique called Short-stump and high-anastomosis pull-through (SHiP) procedure which consists of anterior rectal resection with total mesorectal excision and two-staged delayed ‘‘high’’ coloanal anastomosis without a permanent or temporary ostomy. The SHiP procedure aims to perform a higher anastomosis maintaining a better sphincter contraction, due to the absence of mesocolic tissue inside the anal canal [1]. However, also with this surgical approach some consequences are still present, in particular, the functional ones. Indeed, low anterior resection syndrome (LARS) which comprehends a collection of symptoms including incontinence, frequency, urgency, or feelings of incomplete emptying is one of the most frequent consequences after low anterior resections that do not involve the creation of an ostomy [2]. These alterations may result in some patients opting for a permanent colostomy to avoid these symptoms and, therefore, it remains a highly debated topic that deserves further strategies and investigations. In this context, transanal irrigation (TAI) has been recently introduced as an inexpensive and effective treatment of LARS [3].

The aim of the present study is to demonstrate the role of TAI in the functional treatment of LARS after SHiP procedure in patients with low rectal cancer.

## Methods

Even though the analysis of this study is retrospective and observational, a prospective database of 17 patients with a diagnosis of LARS after a SHiP procedure [4] was maintained from April 2019 to December 2021. This study reports a single-center experience, and it is written according to the Strengthening the Reporting of Observational Studies in Epidemiology (STROBE) statement for cohort studies [5]. All patients who underwent TAI treatment after SHiP procedure for low rectal cancer reaching at least 3 months of follow-up were included in the analysis.

Patients presenting LARS after surgery were evaluated with validated scores and stratified in degrees of severity. The score administered to patients was LARS score to evaluate the presence of functional symptoms and Cleveland Clinic Incontinence score (CCIS), also called Wexner score, to evaluate the presence of fecal incontinence (FI) [6, 7]. LARS score is composed of five questions consisting of three to four answers per each (minimum score = 0; maximum score = 42). The interpretation consists of three categories of severity: from 0 to 20 score, patients are considered to have no LARS, from 21 to 29 minor manifestation of LARS, from 30 to 42 major LARS symptoms.

CCIS has five parameters, each scoring from 0 to 4 according to the frequency (0 = perfect continence and 20 = complete incontinence). Both the questionnaires were administered at first follow-up and after 2 weeks of TAI treatment.

TAI was performed using the Peristeen system^®^ (Coloplast, Humlebaek, Denmark).

Patients enrolled in the study underwent pelvic floor rehabilitation and biofeedback training for the first three months after surgery. After three months, patients were evaluated through the validated scores, and they started performing TAI procedure every day for the first 5 days and then every other day for 2 weeks. At the end of the second week, all patients were visited, and the scores were administered again.

All patients enrolled in the present study participated in the Bowel Rehabilitation Programme (BOREAL) which represents a proactive strategy to assess and treat patients with LARS.

The BOREAL programme [8] consists of five sequential therapeutic steps starting from an approach as conservative as possible and up to surgery. The first one is medical management (steps 0–1), in case of persistence of symptomatology, the patients undergo pelvic floor physiotherapy, biofeedback and transanal irrigation (step 2), sacral nerve neuromodulation (step 3), percutaneous endoscopic cecostomy and anterograde enema (step 4), definitive colostomy (step 5).

Good functional result, both for BOREAL programme and for the present study, was considered to be a LARS score decrease of 20 combined with CCIS decrease of 4. Categorical variables were analyzed and reported as counts and percentages, and as the mean ± SD (range) for continuous normally distributed variables, whereas ordinal categorical variables and continuous non-normally distributed variables were reported as median [interquartile range (IQR)]. The results associated with a *p* value < 0.05 were considered statistically significant.

## Results

Our population sample consisted of 17 patients who underwent SHiP procedure. Four patients were excluded from the present analysis because of a LARS score inferior to 21 at the first assessment, corresponding to no LARS. The population included in the study had a mean age of 67 years old ± 6 (range 54–79) and a moderate severity of symptomatology with a minimum LARS score presentation of 28 and a minimum of CCIS of 8 (Table 1). Most patients were males (9/13;69%).

**Table 1:** Population of the study and outcomes. LARS I and CCIS I = Baseline; LARS II and CCIS II = after transanal irrigation

	Sex	Age	LARS I	CCIS I	LARS II	CCIS II	Follow-up (months)
1	M	64	30	11	4	4	3
2	M	54	28	8	0	1	21
3	M	64	36	12	3	2	12
4	M	63	32	10	0	1	12
5	M	67	34	11	0	0	12
6	M	59	7	9	–	–	–
7	F	65	36	12	7	3	12
8	F	74	39	15	7	6	10
9	M	75	7	9	–	–	–
10	M	66	39	14	30	8	10
11	F	67	38	17	4	3	3
12	M	67	29	14	0	0	8
13	F	72	39	13	0	1	8
14	F	82	13	4	–	–	–
15	M	79	34	17	4	6	3
16	F	73	11	6	–	–	–
17	M	73	36	14	3	2	3

Mean follow-up was 9 months ranging from 3 to 21 months (SD ± 5.02). LARS median value was 36 (IQR = 8) and drastically improved after TAI treatment to 3 (IQR = 3), while CCIS initial median value was 13 (IQR = 6) that became 2 (IQR = 4) at the follow-up visit. Five patients (38%) reached a LARS score of 0 after TAI, meaning no symptoms and a CCIS of 0 or 1.

12 out of 13 (92%) patients had a LARS decrease of 20 combined with a CCIS decrease of 4. Therefore, only one patient did not reach a good functional result with a LARS score improvement of 9 and a CCIS decrease of 6, at 10 months of follow-up.

Two patients had minor anal bleeding related to cannula insertion. In both cases, the bleeding stopped spontaneously and required a suspension of the procedure for approximately 7 days, after which patients resumed TAI. No patient presented additional adverse effects such as abdominal cramps, leakage after irrigation, proctitis, nausea or pain at insertion.

## Discussion

To our knowledge, this is the first trial present in the literature analyzing the safety and the efficacy of TAI after SHiP procedure for low rectal cancer.

Currently, low anterior resection, open or minimally invasive, remains the procedure of choice for patients with low rectal cancer even if the not-negligible rate of post-surgical complications both organic ones like the rate of anastomotic leakage and functional ones regarding the delicate anatomic area of anal sphincters that this surgery directly involves, must be considered.

Traditionally, hand-sewn coloanal anastomosis after ultralow anterior resection with diverting stoma has been the safest and most performed procedure. In 1961, TC proposed a new surgical technique that aimed to spare the sphincter complex with a delayed coloanal anastomosis, also called the “pull-through technique”, avoiding a permanent stoma as first try. However, also this kind of procedure was not without any complications, and it has been abandoned in favor of traditional surgery.

Our modified TC technique does not involve the creation of a diverting ostomy with the potential drawback of not protecting the patient from the morbidity and mortality of an anastomotic leak. However, recently, Biondo et al. reported that the two-staged anastomosis procedure is as safe as the immediate colo-anal anastomosis with diverting ileostomy, with the advantage of avoiding the diversion and its potential associated complications. [9].

It is precisely in this context that TAI procedure finds its place. In fact, the first results of TAI after SHiP procedure, on the population analyzed in this study, appear to be very encouraging. TAI application has proven to be effective and inexpensive in the post-operative period. Furthermore, it appears to be easy for patients to learn after just one session of training. We previously reported similar functional results, in terms of LARS and Wexner score, after SHiP procedures with respect to the results regarding traditional surgery that are currently reported in the literature [10].

Based on these  preliminary results, it may be reasonable to think of TAI also as a preventive measure that can be utilized in this kind of surgery without ostomy. Indeed, TAI is a kind of minimally invasive approach that precedes, and tries to avoid, other operative procedures such as sacral nerve stimulation, antegrade colonic irrigation or stoma formation whose direct and indirect costs for the patient are certainly greater. From the data we recorded, the administration of TAI 3 months after surgery proved to be safe with minimal side effects that resolved spontaneously after a short suspension of treatment. However, the major limitation of this study is represented by the small sample of population enrolled and the limited follow-up.

## Conclusion

The first results after SHiP procedure followed by TAI application are very promising in terms of reduction of LARS score. TAI application in the treatment of LARS may represent a great modification of the quality of life of patients both in the choice of surgical technique, preferring the one without a diverting ostomy, and in the management of functional complications after SHiP procedure.
